# Intelligence Can Be Used to Make a More Equitable Society but Only When Properly Defined and Applied

**DOI:** 10.3390/jintelligence9040057

**Published:** 2021-11-25

**Authors:** LaTasha R. Holden, Sara A. Hart

**Affiliations:** 1 Department of Psychology, The University of Memphis, Memphis, TN 38152, USA; 2 Department of Psychology, Florida State University, Tallahassee, FL 32306, USA; sahart@fsu.edu; 3 Florida Center for Reading Research, Tallahassee, FL 32306, USA

**Keywords:** intelligence, inequity, social issues

## Abstract

In the US, undeniable evidence shows that socioeconomic inequities explain a high proportion of individual differences in school achievement. Although not all countries show this same effect due to socioeconomic status, it is consistently found that social inequities lead to achievement gaps. These achievement gaps then manifest into trajectories that set some individuals on a path of lower incomes, poorer health and higher mortality, lower wellbeing, and other poor adult outcomes. Like James Flynn so handily reminded the scientific literature that achievement gaps are explainable by environmental factors, the inequities we see around the world are based on environments some children are exposed to. In his work, Flynn stated his belief that the suppression of scientific work on intelligence would continue to lead to social inequities. We wish to take this idea and move it forward. We believe that the scientific construct of intelligence plays a key role in helping create a more equitable society through science. We also believe that the poor perception of intelligence, rooted in historical realities, means that it will continue to be misunderstood, feared, and misused, limiting how effective it could be in helping to close gaps in achievement and in creating a more equitable society.

## 1. Introduction

In the US, undeniable evidence shows that social inequities explain a high proportion of individual differences in school achievement. We define social inequities as group differences in access to resources based on race/ethnicity and skin color due to social and structural discrimination. The differences in access to resources contribute to disparities felt by these groups, including achievement gaps. Achievement gaps are when marginalized individuals tend to perform lower on standardized school achievement tests than White individuals (e.g., Jeynes 2015; Norman et al. 2001; Taylor 2006). These achievement gaps then manifest into trajectories that set some individuals on a path of lower incomes, poorer health and higher mortality, lower wellbeing, and other poor adult outcomes (Beck and Muschkin 2012; Caro 2009; Taylor 2006). Here we address the real-world problem of achievement gaps—unexplainable other than by social inequities—and layout our opinion that the scientific construct of intelligence must be considered in helping reduce these achievement gaps, and therefore plays a key role in helping create a more equitable society.

A conventional definition of intelligence is that it represents a person’s ability to adapt to new and changing environments, and by extension, people are thought to vary in this ability. If we replaced “intelligence” with a new term, most people would read the previous sentence and think that term sounds like a useful scientific construct. We understand that for many, the claim that including intelligence in work examining the achievement gap is controversial. The fact is that intelligence is a controversial construct and is often not included in scientific projects, including those on the achievement gap. We believe that this is due to the poor perception of intelligence—rooted in historical realities—which has led to it being misunderstood, feared, and misused, also limiting how effective it could be to work towards a more equitable society. Intelligence as a construct has been left behind by much of scientific research and replaced with more socially favorable constructs that remove historical baggage but do not have the same depth of prediction (e.g., executive function, self-regulation, see Daucourt et al. 2018). With our paper, we wish to propose that the concept of intelligence is too important to lose in our science, but we must first properly define intelligence, recognize the history of its previous application, and consider the appropriate application of intelligence in modern society.

## 2. Defining Our Modern Notion of Intelligence 

In Spearman’s (1904) pioneering work using factor analysis, he claimed that it was possible to not only assess cognitive ability, but that one could do so objectively through a latent factor of ability, which he coined *g*, or the general factor of intelligence. Spearman observed what is now called the positive manifold—the finding that performance on one cognitive ability task is highly positively correlated with performance on other tasks. This remains one of the most highly replicated findings in psychology and psychometric literature (Spearman 1904; Conway and Kovacs 2015). Although Spearman defined intelligence in terms of a single psychometric factor *g*, modern work defines intelligence as the emergent interaction of a variety of domain specific and domain general cognitive processes (see Alfano et al. 2016; Van der Maas et al. 2006; Thomson 1916), deemed Process Overlap Theory (Kovacs and Conway 2016). Here, we focus on this modern definition of intelligence with the formative *g* approach supplied by Process Overlap Theory.

Conway and Kovacs (2015) outline the reflective and formative models of *g*. The idea behind the reflective model is that if someone is higher on *g*, they will have a higher IQ score compared to someone lower on g, and the variance in latent *g* determines the variance in the measure. The reflective model assumes a fixed perspective of ability in that *g* is thought to be responsible for one’s performance as well as the variance we see in other measures. On the other hand, the formative model of *g* implies that *g* emerges from the positive manifold we observe between many measures of cognitive ability. In the formative model, *g* does not cause performance but rather performance on diverse cognitive measures causes *g* to emerge (Conway and Kovacs 2015). Whereas reflective models of intelligence suggest that there are limitations in terms of how much improvement is possible for test performance—based on forms of intervention—the formative model of *g* allows us to refocus the conversation. Instead of viewing *g* as the innate and fixed cause of all intelligent and adaptive behavior, we can refocus on how formative models allow for a better understanding of how to better tailor forms of intervention. We believe that by improving tailoring forms of intervention we can better address modern issues surrounding social inequities and their impact on a variety of future life outcomes. 

Kovacs and Conway (2019) argue that because *g* is emergent, intelligence or IQ is not “real” and should not be viewed as general cognitive ability. Instead, *g* is an index that emerges from several real abilities on a variety of diverse subtests. In line with this, Gottfredson (1997) states that intelligence goes beyond just test scores or test performance, but instead reflects aspects such as “figuring out what to do”. Others argue that intelligence test scores are essentially a representation of what the assessment measures (Farmer et al. 2020; Van der Maas et al. 2014). Thus, we should think more deeply about how the scores on intelligence tests are obtained and what different submeasures of IQ mean, especially for solving real world problems. Based on Kovacs and Conway, there are three levels of evaluation that future research might focus on for intervention purposes: the subtests, the specific factors, and the global scores or the general factor of intelligence. In the formative approach—moving beyond simply exploring the higher order concept of global IQ—investigating specific abilities allows for a more informed understanding of intelligence and where there might be room for intervention. We will unpack this more, but first, we want to briefly consider what the literature finds on the question of whether or not intelligence is improvable.

‘Intelligence as improvable’ is contrary to early definitions and some modern views consider it innate and relatively unchangeable (Jensen 1969, 1998). In terms of ways to improve intelligence, there is evidence showing that performance on some intelligence tests is at least in part malleable and trainable (Binet [1909] 1973; Cury et al. 2008; Gould 1981; Flynn 1984; Jaeggi et al. 2008; also see Jaeggi et al. 2011; 2013; Redick et al. 2013; also see Nisbett et al. 2012). For example, some show that fluid intelligence might be improvable via working memory (see Baddeley and Hitch 1974) training (Au et al. 2015; Jaeggi et al. 2008, although this is not without controversy, e.g., Redick et al. 2013; Shipstead et al. 2012). Some early forms of intervention (Protzko et al. 2013) and education can improve intelligence scores (Ritchie and Tucker-Drob 2018), and individual differences factors underlying intelligence scores change across the lifespan due to contextual factors (Haworth et al. 2010; Tucker-Drob and Bates 2016). Moreover, it has been argued by some that scores on intelligence tests are biased and influenced by cultural differences (Cattell 1949; Williams 1972), which are subject to situational and environmental influences making intelligence “socially situated” (Rogoff and Lave 1984; Walton 2013). These findings bring up important points about the role of context, the person, and the tool, in impacting differences in performance on intelligence measures. Collectively, however, these findings show that performance on tests of intelligence is improvable, a fact that we argue can be useful. 

With a more targeted focus on where there might be room for improvement, Kovacs and Conway (2019) imply that subtests and specific factors that compose a global index of intelligence would prove more useful than focusing on a global index or the general factor of intelligence alone. This is worth considering as an avenue for future research to further examine the efficacy and utility of intelligence to better support marginalized students. Considering the underlying specific abilities that make up global IQ, the specific abilities level is optimal because as Kovacs and Conway state: “it is specific enough to allow for a meaningful reflective interpretation but global enough to not be entirely task specific”. Formative models of *g* provide the benefit of focusing more on lower order specific abilities (i.e., fluid, spatial, and verbal specific abilities) because they are real, and beyond being statistically emergent (like global IQ or *g*), they have predictive validity. This allows us to find ways to improve performance and achievement with intervention, targeting lower-order submeasures of *g*. Based on this, we argue that the formative approach of *g* supplied by Process Overlap Theory has the power to uncover new forms of more tailored intervention and can also help to minimize forms of adverse impact but only when intelligence scores are correctly interpreted and applied as Kovacs and Conway advise. To get to this next step and implement the approach outlined above, we feel that it is important to further examine how the historical realities of intelligence have obstructed its use and application for a more equitable society.

## 3. Issues with Intelligence, and Why They Get in the Way of Equity Goals 

The study and use of intelligence have been marred by its original roots in eugenics (see Harden 2021). Beyond eugenics, throughout the century and a half since intelligence was first conceptualized and measured, it has been used to support the marginalization of racial minorities and impoverished communities (see Beeghley and Butler 1974) and forced the sterilization of individuals with developmental disabilities (Buck v Bell, see Russell 2009). It has been widely misused to support nationalistic and white supremacist agendas by taking the individual differences score measured by an intelligence test and applying it to group differences, such as the so-called “national IQ” (Lynn 2010; also see Wicherts et al. 2010). The consistent finding of moderate heritability on intelligence has been twisted to inaccurately imply biological determinism and immutability of intelligence (see van Dijk et al. 2021, for what heritability means; see also Turkheimer et al. 2003 for a different approach to thinking about heritability). The conceptualization of intelligence was originally rooted in a Western Upper-class European culture, and that does not universally apply to all cultures around the world (e.g., Sternberg et al. 2001). Furthermore, our understanding of intelligence is almost entirely limited to samples that lack racial and ethnic diversity, as well as being “WEIRD,” or White, Educated, Industrialized, Rich, and Democratic (Henrich et al. 2010). We take the stand here acknowledging that diversity and inclusion related concerns as it pertains to research are important. We argue that white nationalistic agendas have contributed to a lack of appropriate representation and ethical participation of marginalized groups in the science of intelligence. This, in turn, has undoubtedly contributed to the construct of intelligence being underappreciated in groups who have traditionally been stigmatized or disenfranchised by definitions and applications of intelligence in the real world. However, by avoiding the construct of intelligence, we have ignored what the construct has to offer in terms of helping to solve a real-world problem related to social inequities: achievement gaps.

## 4. How Can the Modern Notion of Intelligence Address Real World Inequity Problems?

When our modern notion of intelligence, as defined above, is used empirically, it is shown to predict many real-world outcomes—such as everything from better academic performance in school to getting divorced, to the number of offspring, and even the risk of early death (see Ritchie 2015). Certainly, it could be argued that very few constructs predict more useful outcomes than intelligence, and here we argue that modern notions of intelligence including a formative approach with submeasures of *g* allowing users to solve problems related to social inequities existing in achievement gaps. Next, we discuss two specific real-world problems related to intelligence and achievement: the effects of stereotype threat and learning disabilities.

### 4.1. Stereotype Threat

In terms of social inequity and the impact on performance, for some, the stigma associated with belonging to certain group identities could influence additional variation and induce a form of measurement bias when being assessed on tests of achievement (see Wicherts et al. 2005). This may come from worries of being stereotyped based on aspects attributed to certain social or group identities (Steele and Aronson 1995). For example, Steele and Aronson demonstrate the power that a particular social situation can have on cognitive performance. By inducing threat—making some negative aspect of group membership salient—those under threat suffered significantly decreased task performance relative to control groups. Threat has been shown to interfere with attentional control on the anti-saccade task (Jamieson and Harkins 2007), increase anxiety and lower standardized test performance (Steele and Aronson 1995), and deplete cognitive resources in the short and long term (Inzlicht and Schmeichel 2012; Schmader and Johns 2003; also see Schmader et al. 2008). Threat has also been shown to interfere with initial learning and impact later memory for studied information (Taylor and Walton 2011). There is also evidence that threat effects interact with trait measures of working memory for women and minorities (Holden et al. 2020; Regner et al. 2010). 

With the domain general processes of working memory (as a submeasure of intelligence) being shown as a protective factor for cognitive control and performance, it becomes important to examine ways to enhance working memory. As discussed above, it is unclear whether working memory training improves intelligence; however, there is more evidence that working memory itself is improvable through working memory training (Jaeggi et al. 2008; Redick et al. 2013) and mindfulness practices (Mrazek et al. 2013). Forms of mindfulness practices not only provide psychological and physical health benefits (Grossman et al. 2004; Prazak et al. 2012) but have also been shown to combat negative thought processes like task unrelated thoughts and mind wandering while promoting forms of goal maintenance and attentional control processes (Morrison and Jha 2015) which are also important general processes involved in working memory and intelligence (Conway et al. 2003; also see Kane and Engle 2003). Based on Process Overlap Theory (see Kovacs and Conway 2019), further investigating the interaction of a variety of domain general and domain specific processes of intelligence and ways to support or boost performance on submeasures of intelligence like working memory capacity should be beneficial to students who contend with psychologically threatening situations. Taking this one step further, these forms of intervention may help some of our most marginalized students enhance their test performance. In fact, motivated by inconsistencies in the effectiveness of interventions for stereotype threat, recent meta-analytic work by Liu et al. (2021) found that both person and situation centric approaches helped to counter stereotype threat. They show that belief and identity-based interventions helped most, followed by resilience-based ones. Belief-based interventions focus on changing negative beliefs, emphasizing the promotion of positive beliefs and belonging; whereas, identity-based interventions involve activation of positive aspects of one’s identity in order to help counter stereotype threat and its effects. Resilience based interventions (such as those we argue for here) involve activating self-regulatory processes that aid in helping individuals to cope with stereotype threat and its effects in a more adaptive way. Furthermore, future work should further investigate the efficacy of a combination of these intervention approaches. Theoretically, additional forms of support that help marginalized students combat stereotype threat, contributes to better performance trajectories (Cohen et al. 2017; Yeager and Walton 2011), helping to potentially close gaps in achievement and support a variety of improved-life outcomes whether psychologically, physically, and/or economically (see DeWalt et al. 2004).

Some might believe that the approach we are arguing for is deficit based. To those who think that way, let us clearly and emphatically state: that is simply not true. Those who are marginalized in science and society are not to blame for issues of systemic inequities and societal bias. Those who are the most marginalized in society contend with forms of inequity, including prejudice, stereotyping, and discrimination. Instead of viewing this as a deficit of those who are the targets of these issues, we argue that our approach is all about empowering the targets while they navigate systems that are unfair, biased, and oppressive. We believe that forms of changing unfair systems and structures are also important, and we feel that this should happen *in tandem with* empowering those who face many obstacles every day within slow-changing and unfair systems. We acknowledge that forms of social psychological intervention (e.g., Growth Mindset, Social Belonging, and Self Affirmation, see Yeager and Walton 2011) have been aimed at marginalized students and we believe that in addition to that work, more can be done in the area of intelligence to find forms of cognitive support for students as well. Through the approaches we argue for here, we are centering the experiences of the marginalized and working to include them in our science on intelligence in ways that are both empowering and strengths focused. We wish to acknowledge the truth about what marginalized individuals are often forced to contend with while also finding ways to leverage individual strengths to better tailor forms of intervention to support diverse needs. We are arguing that intelligence research can be applied in this way but only if researchers are willing to acknowledge that differences in experiences also means being understanding of and amenable to differences in needs. Moreover, with the participation of the marginalized and most vulnerable being misused for unethical scientific purposes and excluded or underrepresented in the basic science for so long, there should be a sense of urgency for inclusion, justice, and the instantiation of reparative science that allows for greater beneficence for these groups. Next, we turn to the topic of learning disabilities to further consider how intelligence research can be beneficial.

### 4.2. Learning Disabilities

In the US, Black, Hispanic, Asian, Native American, and language-minority children are overrepresented in special education services (see Morgan et al. 2017, 2018 as to whether this represents a discriminatory overrepresentation or an underrepresentation; see also Woods et al. 2021). There is a history of using a reflective notion of intelligence to understand and define learning disabilities. For example, the IQ-achievement discrepancy definition of dyslexia states that dyslexia can only occur when reading performance is below what would be expected from ability, measured as IQ. The idea is that a child with low intelligence would be expected to also struggle to read, and therefore would not have dyslexia, a domain specific disorder, but instead have global struggles. However, the evidence does not support the idea that individuals who only struggle with reading are different than individuals who have lower intelligence and also struggle to read, casting much doubt on the IQ-achievement discrepancy definition (e.g., Toth and Siegel 2020). Rather than using a reflective notion of intelligence, by using a formative model of intelligence, intelligence tests, which are highly reliable measures, can be used to determine a pattern of strengths and weaknesses for each child in special education. This leads to an individual-differences perspective to our identification of learning disabilities and how we instruct children.

There is good reason to believe that fully understanding individual differences in student performance, including the role of intelligence and its mechanisms, will help us better identify students who will struggle and differentiate our instruction to meet the needs of every student (Connor et al. 2009; Cronbach and Snow 1977; Fuchs and Fuchs 2018). For example, executive function is a good indicator of reading difficulties (Daucourt et al. 2018), suggesting that mechanisms of intelligence might be good indicators of hybrid models of learning disabilities (e.g., Fletcher et al. 2013; Wagner 2008). Phonological awareness, rapid naming speed, alphabetic-principle knowledge, reading skill, and memory capacity have all also been shown to be important individual differences predictors of which children will respond to educational interventions for at risk or struggling readers (Al Otaiba and Fuchs 2002; Coyne et al. 2018; Hart et al. 2016). Considering this, we argue that precision education is an intervention approach (Hart 2016) motivated by the idea that different students have different needs and that we should be aiming to uncover how to better tailor forms of intervention.

## 5. A Thorny Problem and Conclusions

We admit that we were high-level in our discussion of how intelligence can be applied to help close achievement gaps, specifically considering the effects of stereotype threat and the identification and treatment of learning disabilities. We conclude here by discussing standardized testing, which is a thorny real-world problem where thinking about formative intelligence can be useful in some ways of better understanding achievement gaps, but not all.

Recently, there has been an uptick in conversation about the utility of standardized testing and whether they contribute to bias in the academic admissions process (see Michel et al. 2019). There are layers to this conversation that must be distinguished for the sake of clarity. First, some believe that standardized tests like the SAT and GRE (often viewed as synonymous with intelligence) are inherently biased such that they favor White and Asian test-takers and higher SES persons over minority students and lower SES persons. In turn, some argue that standardized tests and particularly the GRE should be optional or potentially abandoned. We will tackle the issue of test bias with two main points. First, the topic of bias in standardized testing has been addressed by the College Board and the Educational Testing Service (ETS) which works to create and administer the SAT and GRE. ETS ensures that their tests are corrected for potential bias from the item level through the entire assessment considering the diversity and multicultural nature of their client-base (Educational Testing Service 2018, Fairness Guidelines), so these tests are unbiased, statistically speaking. Second, where standardized tests are concerned, the biggest problem with inequality stems from access to preparation materials and differences in socioeconomic status that influence access and preparation. As such, these kinds of barriers can mean that these tests are rooted in the inequality that is inherent in society. From the formative perspective, submeasures and subtests are not able to remove or account for forms of social bias inherent in society that might “unlevel the playing field” and impact achievement. As of yet, intelligence tests, like many tests, do not statistically control for these types of differences (we refer the reader to Hartocollis 2019 on the College Board’s proposed privilege and adversity index and its subsequent retraction). This means intelligence is not, nor should it be, thought of as the “end all, be all”. As such, we must keep intelligence scores, like other test scores, in context and be thoughtful about what these scores tell us and consider the limitations of what they cannot tell us^1^.

When thinking more critically about leveling the playing field, we believe that standardized tests, an exemplar of intelligence tests, *can* offer a way to consistently evaluate what we believe to be statistically unbiased measures of cognitive abilities but only to the extent that intelligence is properly understood, defined, and applied. On the contrary to arguments about bias in standardized tests and intelligence measures, we argue that testing such as this *can* give students from disadvantaged backgrounds the chance to stand out and overcome disadvantages^2^. For example, we believe that through a formative *g* approach with Process Overlap Theory, we can generate and test new and meaningful hypotheses about how to combat issues of inequities in education and achievement. Through science, we can then develop more tailored forms of intervention to meet a diversity of needs. In the end, our society is ever-evolving. It seems obvious that a measure of adaptability to a changing environment should be important to our science and our society. As intelligence researchers, we must identify and reconcile the ghastly history surrounding intelligence, we must relegate the nefarious actors who wish to twist intelligence into a discriminatory tool to the corner, and we must thoughtfully research how intelligence can help us towards an equitable society. Like Flynn (1999) so handedly reminded the scientific literature that achievement gaps are explainable by environmental factors, the inequities we see around the world are based on environments some children are exposed to. In the same work, Flynn stated his belief that the suppression of scientific work on intelligence would continue to lead to social inequities. By simply ignoring intelligence or replacing it with a less precise but more fashionable construct, we are ignoring a key tool in cultivating a more equitable society.

## Notes

1 There have been recent debates on the related topic of going ‘test optional’. We argue that making standardized tests optional, or even abandoning them, is not necessarily better for reducing bias and inequality in the admissions process overall. With going ‘test optional’, the question becomes: how do administrators compare students who are similar in grades and experience when one has a standardized test score and one does not? Further, if the students both have stellar letters of recommendation, should the standardized test score for one student be ignored? What forms of bias would this process introduce that have not been previously considered? Moreover, under circumstances of completely abandoning test scores, administrators must rely even more heavily on letters of reference in addition to grades, and we know that forms of bias and inequality are baked into those as well (Miller et al. 2021; Morgan et al. 2013; also see Kumar and Hamer 2013). For example, teachers and administrators are themselves biased (Haughbrook 2020; also see Riddle and Sinclair 2019).

2 As an example, we note the finding that minority and lower SES students are less likely to be identified and recommended for gifted programs (Research News @Vanderbilt 2016). It has been estimated that over 3.6 million students in the US should be labeled as gifted who are not, and this is thought to be the case because minority and lower SES students are largely ignored and not encouraged by their instructors (Hechinger Report 2019; Grissom and Redding 2016). Some have found that underperformance of minority students is driven by greater levels of implicit bias in teachers, and this effect has been explained by greater levels of anxiety and lower levels of lesson quality (Jacoby-Senghor et al. 2016). On the other hand, implementation of universal screening programs (which involve the administration of cognitive aptitude tests), apart from parent and teacher referrals, alone found substantial increases in those identified for giftedness from disadvantaged backgrounds, minority students, and traditionally underrepresented students (Card and Giuliano 2016).
